# Mutant huntingtin disturbs circadian clock gene expression and sleep patterns in *Drosophila*

**DOI:** 10.1038/s41598-019-43612-w

**Published:** 2019-05-09

**Authors:** Anikó Faragó, Nóra Zsindely, László Bodai

**Affiliations:** 1 Department of Biochemistry and Molecular Biology, Faculty of Science and Informatics, University of Szeged, 6726 Közép fasor 52, Szeged, Hungary; 2 Doctoral School in Biology, Faculty of Science and Informatics, University of Szeged, 6726 Szeged, Hungary

**Keywords:** Gene regulation, Huntington's disease, Circadian regulation

## Abstract

Deficiency of the sleep-wake cycle can accelerate the progression of Huntington’s disease (HD) and exacerbate symptoms making it a target of investigation to better understand the molecular pathology of the disorder. In this study we analyzed sleep defects in a *Drosophila* model of HD and investigated whether disturbed sleep coincides with alterations in the molecular mechanism controlling circadian rhythm. To analyze sleep defects we recorded the daily activity of flies in 12:12 hours light:dark entrainment and in regard to the underlying molecular mechanism measured circadian “clock” gene expression. In HD flies we observed reduced amount of sleep, sleep fragmentation and prolonged sleep latency. We found changes in gene expression patterns of both transcriptional feedback loops of circadian regulation. We detected prolonged expression of the core feedback loop components *period* and *timeless*, whilst the secondary feedback loop member *vrille* had lower expression rates in general. Our results show that the *Drosophila* HD model recapitulates most of the sleep related symptoms reported in patients therefore it can be a potential tool to study the molecular background of sleep defects in HD. Altered expression of circadian “clock” genes suggests that disturbed sleep pattern in HD might be the consequence of disturbed circadian regulation.

## Introduction

Circadian rhythm disruption and consequent sleep abnormality is a common feature of the most prevalent neurodegenerative diseases, such as Alzheimer’s disease and Parkinson’s disease, and recently it was also reported in Huntington’s disease^1–3^. HD patients have disturbed nocturnal sleep with prolonged sleep-onset latency, fragmented and irregular sleep stages and increased wakefulness^4–7^. Sleep disruption can lead to severe symptoms (depression, aggressivity, impaired memory and executive control)^8^, several of which are also present in HD^9,10^. Furthermore, sleep loss can accelerate the progression of the disease^11^, therefore understanding the defects of the underlying molecular mechanisms are essential.

HD is a late onset neurodegenerative disorder caused by abnormal expansion of a glutamine coding CAG repeat in the first exon of the *Huntingtin* (*HTT*) gene encoding the huntingtin protein^12^. Transcriptional dysregulation is one of the earliest pathological alterations in HD^13,14^. As circadian rhythm is regulated by transcriptional feedback loops^15^ it is feasible that the transcriptional defect in HD might affect the circadian clock as well.

In *Drosophila* circadian timekeeping is regulated by two interlocking feedback loops (core and secondary loop) that maintain the oscillating expression of “clock” genes in pacemaker neurons^15^. dCLOCK/CYCLE (dCLK/CYC) heterodimer regulates the transcription of *period* (*per*), *timeless* (*tim*), *vrille* (*vri*), *Par domain protein 1* (*Pdp1*) and *clockwork orange* (*cwo*)^16–18^. As part of the core feedback loop DOUBLETIME/PER/TIM complex inhibits the activity of dCLK/CYC^19^ subsequently repressing their own transcription. In the secondary feedback loop CWO inhibits the activity of dCLK/CYC^18^, while VRI represses and PDP1 activates the expression of the *dClk* gene itself^17^. As a result of the feedback regulation the expression of *per*, *tim*, *vri*, *Pdp1* and *cwo* is low at dawn and peaks at dusk, however, that of *dClk* is antiphase with high expression in the morning and low expression in the evening^15^. Defects of regular cycling of “clock” gene expression lead to disruption of the circadian rhythm^20,21^.

In this study we present a *Drosophila* model of HD displaying sleep defects similar to those described in patients. Furthermore, we demonstrate dysregulated expression of certain circadian “clock” genes in both feedback loops.

## Materials and Methods

### *Drosophila* melanogaster stocks and crosses

Stocks were maintained and crosses were done on standard *Drosophila* medium. To model HD we used flies that express human *HTT* exon 1 with 25 (*HTTex1Q25*, control) or 120 (*HTTex1Q120*, mutant) glutamines under the control of yeast Upstream Activating Sequence (UAS). *w*; *UAS*-*HTTex1Q25* and *w*; *UAS*-*HTTex1Q120* strains were donations of J. Lawrence Marsh (University of California Irvine, USA)^22^. *w P*{*GawB*}*elav*^*C155*^ (henceforth *elavGAL4*), *y*^*1*^ w^*^; *P*{*w*^+*mC*^ = *GAL4*-*per*.*BS*}*3* (henceforth *perGAL4*), and *P*{*tubP*-*GAL80*^*ts*^}*7* (henceforth *tubGAL80*^*ts*^) lines were from the Bloomington *Drosophila* Stock Center. For activity recordings the expression of *HTT* transgenes was achieved by the *elavGAL4*; *tubGAL80*^*ts*^ temperature sensitive pan-neuronal driver combination. Crosses of *elavGAL4*; *tubGAL80*^*ts*^ females and *UAS*-*HTTex1Q25* or *UAS*-*HTTex1Q120* males were done at 18 °C, F1 males were transferred to 30 °C after eclosion to induce transgene expression. This expression system improves the validity of the disease model as it mimics the adult onset of HD. For gene expression analysis we used the *perGAL4* driver at 25 °C that directs transgene expression in the pattern of the *period* gene.

### Activity recording and analysis

**Figure 1:**
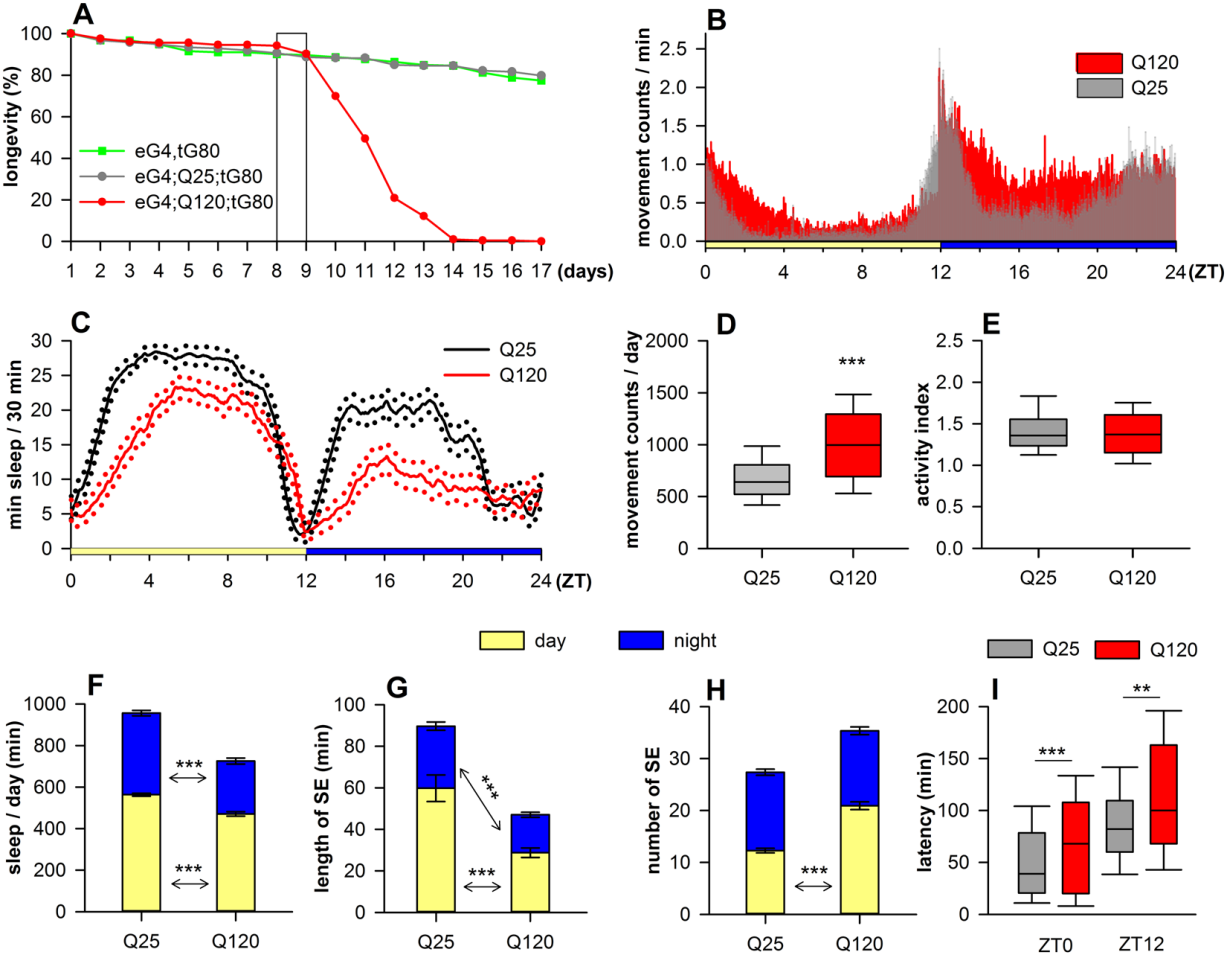
Sleep pattern and daily activity is disturbed in *HTTex1Q120* expressing flies. (**A**) Longevity analysis shows that the lifespan of *elavGAL4*/*Y*; *UAS*-*HTTex1Q120*/+; *tubGAL80*^*ts*^/+ (eG4;Q120;tG80) flies is reduced compared to *elavGAL4*/*Y*; *UAS*-*HTTex1Q25*/+; *tubGAL80ts*/+ (eG4;Q25;tG80) flies or driver only controls (eG4;tG80). Survival of *HTTex1Q120* flies starts to rapidly decline on day 9, therefore activity measurements started on day 8 from 8:00 in the morning until day 9 8:00 in the morning (outlined area). (**B**) Daily locomotor activity of 8 days old flies was measured in 12:12 light:dark entrainment. *Drosophila* is mainly active at dawn (ZT0) and at dusk (ZT12), while less active in between. HD flies (Q120) show elevated activity both in light and dark compared to *HTTex1Q25* expressing controls (Q25). (**C**) In parallel with increased motor activity HD flies spend less time asleep than controls (solid lines represent the averages, dotted lines show 95% confidence intervals). (**D**) In HD flies the number of total daily movement counts was increased. (**E**) However, there was no change in the activity index – total movement counts normalized to the time spent awake. (**F**) This is explained by the finding that the total amount of time HD flies spend asleep was decreased both daytime (yellow) and nighttime (blue). (**G**) The length of sleep episodes decreased during both daytime and nighttime, however, (**H**) the average number of sleep episodes increased only during daytime. (**I**) The onset of both daytime and nighttime resting periods was prolonged in *HTTex1Q120* expressing flies. Activity was recorded with DAM2 *Drosophila* Activity Monitor over a period of 24 hours (ZT0 = lights on, ZT12 = lights off). Data are plotted as mean ± SEM (*HTTex1Q25* n = 107, *HTTex1Q120* n = 111). **P < 0.01, ***P < 0.001, Student’s *t*-test.

*elavGAL4*/*Y*; *UAS*-*HTTex1Q25*/+; *tubGAL80*^*ts*^/+ and *elavGAL4*/*Y*; *UAS*-*HTTex1Q120*/+; *tubGAL80*^*ts*^/+ males that eclosed in a 24 hour time period were transferred to fresh vials and placed to 30 °C to induce *HTT* expression. Flies were synchronized and entrained by exposing them to 12:12 hours light (~250 lx):dark (LD) cycles for 7 days before performing the activity recordings with DAM2 *Drosophila* Activity Monitor (TriKinetics Inc, Waltham, MA, USA) that records the activity of 32 individual flies simultaneously. We recorded the daily activity of 8 days old *HTTex1Q25* (n = 107) and *HTTex1Q120* (n = 111) flies over a period of 24 hours starting from ZT0 (Zeitgeber Time: refers to time in hours during a light-dark cycle where ZT0 = lights on and ZT12 = lights off). *elavGAL4*/*Y*; *UAS*-*HTTex1Q120*/+; *tubGAL80*^*ts*^/+ males show circadian rhythm defects after 7 days, therefore 24 hours measurements started on day 8 from 8:00 in the morning until day 9 8:00 in the morning, just before flies start to die (Fig. 1A). Data were collected with DAMSystem3 for Windows and analyzed using pySolo analysis software^23^ and by Excel functions. All data are presented as mean ± standard error of mean (SEM). For statistical analysis all parameters were compared by Student’s *t*-test or one-way ANOVA with Tukey HSD post-hoc test.

### Gene expression analysis

Total RNA was isolated from heads of 14-day-old 12:12 hours LD entrained *y w*/*Y*; *perGAL4*/*UAS*-*HTTex1Q25* and *y w*/*Y*; *perGAL4*/*UAS*-*HTTex1Q120* males (at least 3 biological replicates per sampling time, 20 males per replicate) using Trizol Reagent (Invitrogen). RNA concentration and purity were determined by spectrophotometric measurement with NanoDrop® ND-1000 instrument. After DNaseI (Thermo Scientific) treatment cDNA was prepared from 400 ng total RNA using TaqMan^TM^ Reverse Transcription Reagents (Thermo Scientific) with random hexamer primers following the recommendations of the manufacturer. The resulting cDNA was diluted 1:5 and used for qPCR by the SYBR green method with Luminaris Color HiGreen qPCR Master Mix in a PikoReal Real-Time PCR System (Thermo Scientific). qPCR data were normalized first to *Alpha*-*tubulin at 84B* (CG1913) housekeeping gene, then to the corresponding ZT0 sample. For statistical analysis of expression values Two-Way ANOVA (TWA) with Tukey HSD post-hoc test was performed. Amplitude (maximum distance from the mean value) and acrophase (phase of the maximal value change assumed by the curve) was determined using the Cosinor application^24^, and statistically tested using Mann-Whitney U-test (MWU-test).

## Results and Discussion

### HD flies exhibit sleep defects

*Drosophila* is mainly active at dawn (lights on) and at dusk (lights off), while less active in between^23^. We recorded the daily activity of 8-day-old males expressing *HTTex1Q120* (HD) or *HTTex1Q25* (controls) in adult neurons (Fig. 1A,B). The daytime nap of males enables the analysis of two resting periods per day (Fig. 1C). HD flies displayed considerable hyperactivity compared to control (total daily movement counts 689 ± 28 versus 1003 ± 37 in *HTTex1Q25* and *HTTex1Q120*, respectively, P = 1.3652 × 10^−10^) (Fig. 1B,D), while the amount of total daily sleep decreased (956 ± 14 versus 725 ± 18 minutes in *HTTex1Q25* and *HTTex1Q120*, respectively, P = 1.1102 × 10^−16^) (Fig. 1C,F). Comparison of activity indexes - total activity normalized to the total time spent awake - showed no difference (Fig. 1E), meaning that the observed hyperactivity is the consequence of reduced sleep instead of increased motor activity. The decrease in time spent asleep is apparent during both resting periods (daytime sleep: 563 ± 7 versus 471 ± 11 minutes, P = 4.3059 × 10^−11^; nighttime sleep: 393 ± 13 versus 255 ± 15 minutes, P = 1.6046 × 10^−11^ in *HTTex1Q25* and *HTTex1Q120*, respectively) (Fig. 1F).

By analyzing sleep episodes (SE) we found that in case of HD flies the length of daytime SE was shorter (60 ± 6 versus 29 ± 2 minutes in *HTTex1Q25* and *HTTex1Q120*, respectively, P = 6.3321 × 10^−5^) while their number increased (12 ± 1 versus 21 ± 1 in *HTTex1Q25* and *HTTex1Q120*, respectively, P = 1.1102 × 10^−16^) (Fig. 1G,H). The length of nighttime SE was also shorter (30 ± 2 versus 18 ± 1 minutes in *HTTex1Q25* and *HTTex1Q120*, respectively, P = 9.9646 × 10^−7^), however, their number did not change significantly (15 ± 1 versus 14 ± 1 in *HTTex1Q25* and *HTTex1Q120*, respectively) (Fig. 1G,H).

When studying the onset of resting periods we observed that sleep latency – time passing between lights ON/OFF and the start of the first sleep episode - is prolonged in HD flies (daytime latency: 47 ± 4 versus 67 ± 6 minutes, P = 7.3 × 10^−4^, nighttime latency: 81 ± 4 versus 101 ± 7 minutes, P = 3.8 × 10^−3^, in *HTTex1Q25* and *HTTex1Q120*, respectively) (Fig. 1I). To exclude the possibility that phenotypic differences observed between flies expressing *HTTex1Q25* or *HTTex1Q120* are due to inherent differences of the two parental UAS strains we analyzed their circadian activity and sleep phenotypes and found no differences (Supplementary Fig. 1).

Thus, we found that the *Drosophila* model used in this study displays sleep defects characteristic for HD patients including increased daily activity as a consequence of reduced overall sleep, fragmented sleep and prolonged sleep-onset latency. Although sleep defects were reported in animal models of HD previously, none of these presented all the above described abnormalities together^25–27^. A mouse model showed disturbed night-day activity accompanied by disruption of circadian “clock” gene expression, however, mice are nocturnal thus their activity profile is different from that of humans^25,28^. The diurnal OVT73 sheep model displayed evening restlessness and nighttime sleep disturbances that mirror sleep defects of HD patients^26^. Nighttime sleep disruption was also observed in a *Drosophila* model previously. Neuronal expression of an N-terminal HttQ128 fragment from embryogenesis on resulted in reduced locomotor activity and increased daytime and decreased nighttime sleep. Circadian rhythm defects more similar to what we have found were observed in a full-length HttQ128 model: reduced nighttime sleep with more but shorter sleep episodes^27^. However, its molecular background was not investigated, urging us to determine whether disturbed sleep coincides with altered expression of circadian “clock” genes.

### Circadian “clock“ gene expression is altered in HD flies

Understanding the underlying molecular background of sleep disruption in HD may lead to development of better treatment options and improved life quality. Defects in the normal cycling of “clock” gene expression cause the disruption of circadian rhythm^20,21^ suggesting that disturbed sleep-wake patterns observed in HD might be the consequence of inappropriate regulation of “clock” gene transcription.

**Figure 2:**
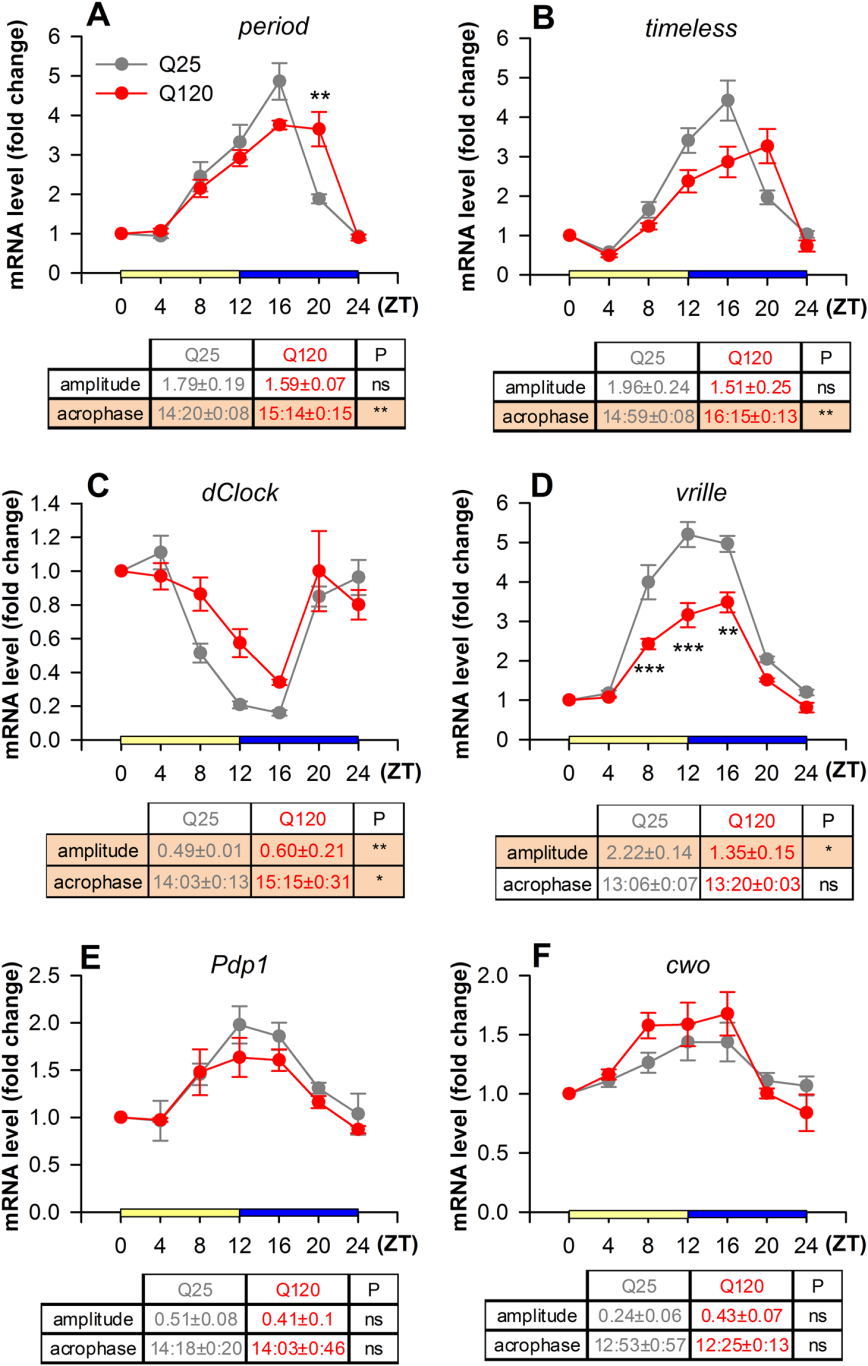
Circadian “clock” gene expression is impaired in flies expressing *HTTex1Q120* under the control of *perGAL4*. Expression of *per* (**A**) and *tim* (**B**) was moderately decreased during ZT8-16, however, at ZT20 it remained elevated (*per* genotype/time interaction P = 6.45 × 10^−4^; *tim* genotype/time interaction P = 1.04 × 10^−3^, TWA). The phase of the maximal change in mRNA level compared to ZT0 (acrophase) was delayed in case of both *per* (P = 0.009, t-test) and *tim* (P = 0.003, MWU-test). *dClk* (**C**) transcript levels were elevated in HD flies (genotype/time interaction P = 0.042, TWA) with significant difference in amplitude (P = 0.0012, MWU-test) and its acrophase was delayed (P = 0.043, MWU-test). Expression of *vri* (**D**) was significantly lower between ZT8-16 in *HTTex1Q120* expressing flies (genotype/time interaction P = 5.58 × 10^−4^, TWA), and the amplitude of its expression was also significantly lower (P = 0.016, MWU-test). Expression of *Pdp1* (**E**) and *cwo* (**F**) did not change significantly (*Pdp1* genotype/time interaction P = 0.925; *cwo* genotype/time interaction P = 0.512, TWA). mRNA levels are shown in fold change normalized to ZT0 sample. Data are plotted as mean ± SEM (n ≥ 3); **P < 0.01, ***P < 0.001, TWA with Tukey HSD test. In the data tables amplitude (fold change values) and acrophase (hours:minutes) are given as mean ± SEM. P columns show the results of MWU-test (*P < 0.05, **P < 0.01, ns: P > 0.05), significant differences are emphasized by orange background.

We investigated whether mutant huntingtin alters the expression of circadian “clock” genes. We analyzed the expression pattern of *dClk*; *per* and *tim* core feedback loop genes; and *vri*, *Pdp1* and *cwo* secondary feedback loop genes by quantitative RT-PCR. Gene expression patterns of the control were congruent with previous findings. The expression of *per*, *tim*, *vri*, *Pdp1* and *cwo* was low at dawn (ZT0) and peaked at dusk (ZT12), while that of *dClk* was the highest in the morning and lowest in the evening (Fig. 2). In HD flies we observed a phase shift in the transcript levels of core feedback loop genes *per* and *tim*, as their acrophase was significantly delayed (P = 0.009 and P = 0.003, respectively) (Fig. 2A,B). Their expression levels were moderately lower between ZT8-16 than in control. However, at ZT20 when *per* and *tim* expression started to drop in the control it remained remarkably high in HD flies (P = 2.4 × 10^−3^ for *per*, whereas for *tim* the difference was not statistically significant). The expression level of *dClk* was higher (P = 0.042) with significant difference in amplitude (P = 0.0012) and its acrophase was delayed (P = 0.043) in HD flies compared to control, however differences at specific timepoints did not reach statistical significance (Fig. 2C). Among the secondary feedback loop genes *vri* showed significantly lower amplitude (P = 0.016) and expression between ZT8-16 in HD flies compared to control (ZT8: P = 1.1358 × 10^−3^; ZT12: P = 1.42 × 10^−5^; ZT16: P = 7.448 × 10^−4^) (Fig. 2D), while the expression pattern of *Pdp1* and *cwo* did not change (Fig. 2E,F).

Thus, we found that the rhythm of circadian “clock” gene expression in heads of flies expressing mutant Huntingtin in the pattern of the *period* gene is distorted but does not disappear. As *perGAL4* drives expression not only in neuronal clusters but also in glial cells and peripheral tissues^29^ the observed gene expression changes reflect the cumulative effects of mutant Huntingtin in these tissues. Importantly, we found altered expression patterns of components of both the core and secondary circadian feedback loops. The dCLK transcription factor, whose activity is primarily regulated on a post-translational level^19^ plays an essential role in the regulation of genes of both loops^15^. In HD flies the mRNA level of VRI, a negative regulator of *dClk* transcription, was lower between ZT8-16, suggesting that it may be responsible for the increased transcription of *dClk*. Prolonged expression of core feedback loop components *per* and *tim* might lead to extended presence of higher levels of PER and TIM proteins and consequent extended inactive state (enacted by the DOUBLETIME/PER/TIM complex) and delayed accumulation of active dCLK. Prolonged presence of phosphorylated, inactive dCLK might lead to suppression of its target genes. If the inactive state of dCLK is extended that might explain the observed lower expression level of *vri*, *per* and *tim* between ZT8-16. Furthermore, delayed accumulation of active dCLK could lead to the observed prolonged expression of *per* and *tim*.

The connections between the molecular circadian clock system and animal behavior are not clearly elucidated, therefore it is not evident how the observed “clock” gene expressional changes lead to the specific sleep defects observed in HD flies. Delayed acrophase of *per* and *tim* in HD flies correlates with delayed nighttime sleep (longer latency at ZT12), however, whether this is a direct or indirect effect is yet to be decided. Altered *vri* expression in HD flies might also affect the expression of circadian clock controlled output genes that might play a role in organism level sleep behavior. A large number of genes whose expression peaks at dawn were identified as potential transcriptional targets of secondary feedback loop components in microarray experiments^30–33^. Therefore, it is feasible that the observed sleep disturbances are the consequence of alterations in secondary loop regulated output gene expression, however, the real underlying molecular background is yet to be clarified.

We find it important that not all “clock” genes were dysregulated in HD flies, the transcription of *cwo* and *Pdp1* secondary loop components did not change. This shows that the observed circadian clock defects are gene specific and are not a consequence of generic transcriptional dysregulation, suggesting that other transcriptional factors or cofactors beside dCLK might be also important in the regulation of these genes.

## Conclusion

We present a *Drosophila* model of HD featuring most of the characteristic sleep-wake disturbance phenotypes observed in HD patients including reduced overall sleep, fragmented sleep and prolonged sleep-onset latency. By analyzing circadian “clock” gene expression patterns in the HD model we found significant differences in the expression levels of the core feedback loop genes *per* and *tim*, and the secondary feedback loop member *vri*. Our findings suggest that the observed sleep disturbances might be the consequence of disordered regulation of genes controlling circadian rhythm. Although the anatomical and physiological differences between insects and mammals set limitations to the use of circadian rhythm studies in *Drosophila* models of human disorders our results show that the *Drosophila* HD model can be suitable to analyze the role of circadian transcriptional regulatory circuits in mutant huntingtin induced circadian and sleep disturbances.

## Supplementary information

Supplementary Figure S1

## Data Availability

The datasets used and/or analysed during the current study are available from the corresponding author on reasonable request.

## References

[1] Musiek ES, Xiong DD, Holtzman DM (2015). Sleep, circadian rhythms, and the pathogenesis of Alzheimer Disease. Exp. Mol. Med..

[2] Li S, Wang Y, Wang F, Liu LHC (2017). A New Perspective for Parkinson’s Disease: Circadian Rhythm. Neurosci. Bull..

[3] Morton, A. J. Circadian Dysfunction in Huntington’s Disease. In *Circadian Medicine* (ed. Christopher, S. C.) 305–320 (John Wiley & Sons, Inc, 2015).

[4] Hansotia P, Wall R, Berendes J (1985). Sleep disturbances and severity of Huntington’s disease. Neurology.

[5] Wiegand M (1991). Nocturnal sleep in Huntington’s disease. J. Neurol..

[6] Goodman Anna O. G., Morton A. Jennifer, Barker Roger A (2010). Identifying sleep disturbances in Huntington’s disease using a simple disease-focused questionnaire. PLoS Currents.

[7] Goodman AOG (2011). Asymptomatic Sleep Abnormalities Are a Common Early Feature in Patients with Huntington’s Disease. Curr. Neurol. Neurosci. Rep..

[8] Orzeł-gryglewska J (2010). Consequences of sleep deprivation. Int. J. Occup. Med. Environ. Health..

[9] Aziz NA, Anguelova GV, Marinus J, Lammers GJ, Roos RAC (2010). Sleep and circadian rhythm alterations correlate with depression and cognitive impairment in Huntington’s disease. Park. Relat. Disord..

[10] Morton AJ (2013). Circadian and sleep disorder in Huntington’s disease. Exp. Neurol..

[11] Musiek ES (2015). Circadian clock disruption in neurodegenerative diseases: cause and effect?. Front. Pharmacol..

[12] The Huntington’s Disease Collaborative Research Group (1993). A novel gene containing a trinucleotide repeat that is expanded and unstable on Huntington’s disease chromosomes. Cell.

[13] Sugars KL, Rubinsztein DC (2003). Transcriptional abnormalities in Huntington disease. Trends Genet..

[14] Moumné L, Betuing S, Caboche J (2013). Multiple aspects of gene dysregulation in Huntington’s disease. Front. Neurol..

[15] Hardin PE (2011). Molecular genetic analysis of circadian timekeeping in *Drosophila*. Adv. Genet..

[16] Allada R, White NE, So WV, Hall JC, Rosbash M (1998). A mutant *Drosophila* homolog of mammalian clock disrupts circadian rhythms and transcription of period and timeless. Cell.

[17] Cyran SA (2003). vrille, Pdp1, and dClock Form a Second Feedback Loop in the *Drosophila* Circadian Clock. Cell.

[18] Kadener S, Stoleru D, Mcdonald M, Nawathean P, Rosbash M (2007). Clockwork Orange is a transcriptional repressor and a new *Drosophila* circadian pacemaker component. Genes Dev..

[19] Yu W, Zheng H, Houl JH, Dauwalder B, Hardin PE (2006). PER-dependent rhythms in CLK phosphorylation and E-box binding regulate circadian transcription. Genes Dev..

[20] Sehgal A, Price JL, Man B, Young MW (1994). Loss of Circadian Behavioral Rhythms and per RNA Oscillations in the *Drosophila* Mutant timeless. Science..

[21] Allada R, Kadener S, Nandakumar N, Rosbash M (2003). A recessive mutant of *Drosophila* Clock reveals a role in circadian rhythm amplitude. EMBO J..

[22] Barbaro BA (2015). Comparative study of naturally occurring Huntingtin fragments in *Drosophila* points to exon 1 as the most pathogenic species in Huntington’s disease. Hum. Mol. Genet..

[23] Gilestro GF (2012). Video tracking and analysis of sleep in *Drosophila* melanogaster. Nat. Protoc..

[24] Refinetti R, Lissen GC, Halberg F (2007). Procedures for numerical analysis of circadian rhythms. Biol. Rhythm. Res..

[25] Morton AJ (2005). Disintegration of the Sleep-Wake Cycle and Circadian Timing in Huntington’s Disease. J. Neurosci..

[26] Morton AJ (2014). Early and progressive circadian abnormalities in Huntington’s disease sheep are unmasked by social environment. Hum. Mol. Genet..

[27] Gonzales Erin, Yin Jerry (2010). Drosophila Models of Huntington's Disease Exhibit Sleep Abnormalities. PLoS Currents.

[28] Kudo T (2011). Dysfunctions in circadian behavior and physiology in mouse models of Huntington’s disease. Exp. Neurol..

[29] Kaneko M, Hall JC (2000). Neuroanatomy of cells expressing clock genes in *Drosophila*: transgenic manipulation of the period and timeless genes to mark the perikarya of circadian pacemaker neurons and their projections. J. Comp. Neurol..

[30] Claridge-Chang A (2001). Circadian regulation of gene expression systems in the *Drosophila* head. Neuron.

[31] Ceriani MF (2002). Genome-wide expression analysis in *Drosophila* reveals genes controlling circadian behavior. J. Neurosci..

[32] McDonald MJ, Rosbash M (2001). Microarray analysis and organization of circadian gene expression in *Drosophila*. Cell.

[33] Wijnen H, Naef F, Boothroyd C, Claridge-Chang A, Young MW (2006). Control of daily transcript oscillations in *Drosophila* by light and the circadian clock. PLoS Genet..

