# Supplementary material for: Mutant huntingtin disturbs circadian clock gene expression and sleep patterns in Drosophila
Source: Sci Rep. 2019 May 9;9:7174. doi: 10.1038/s41598-019-43612-w (PMC6509128; doi:10.1038/s41598-019-43612-w)
Supplement: Supplementary file 1 — Supplementary Figure S1 [file 41598_2019_43612_MOESM1_ESM.pdf]

SUPPLEMENTARY MATERIAL

Mutant huntingtin disturbs circadian clock gene expression and sleep patterns in *Drosophila*

Anikó Faragó[1,2], Nóra Zsindely[1], László Bodai[1,*]

Affiliations

[1]: Department of Biochemistry and Molecular Biology, Faculty of Science and Informatics, University of Szeged, 6726 Szeged Közép fasor 52. Hungary

[2]: Doctoral School in Biology, Faculty of Science and Informatics, University of Szeged, 6726 Szeged, Hungary

*: corresponding author: bodai@bio.u-szeged.hu

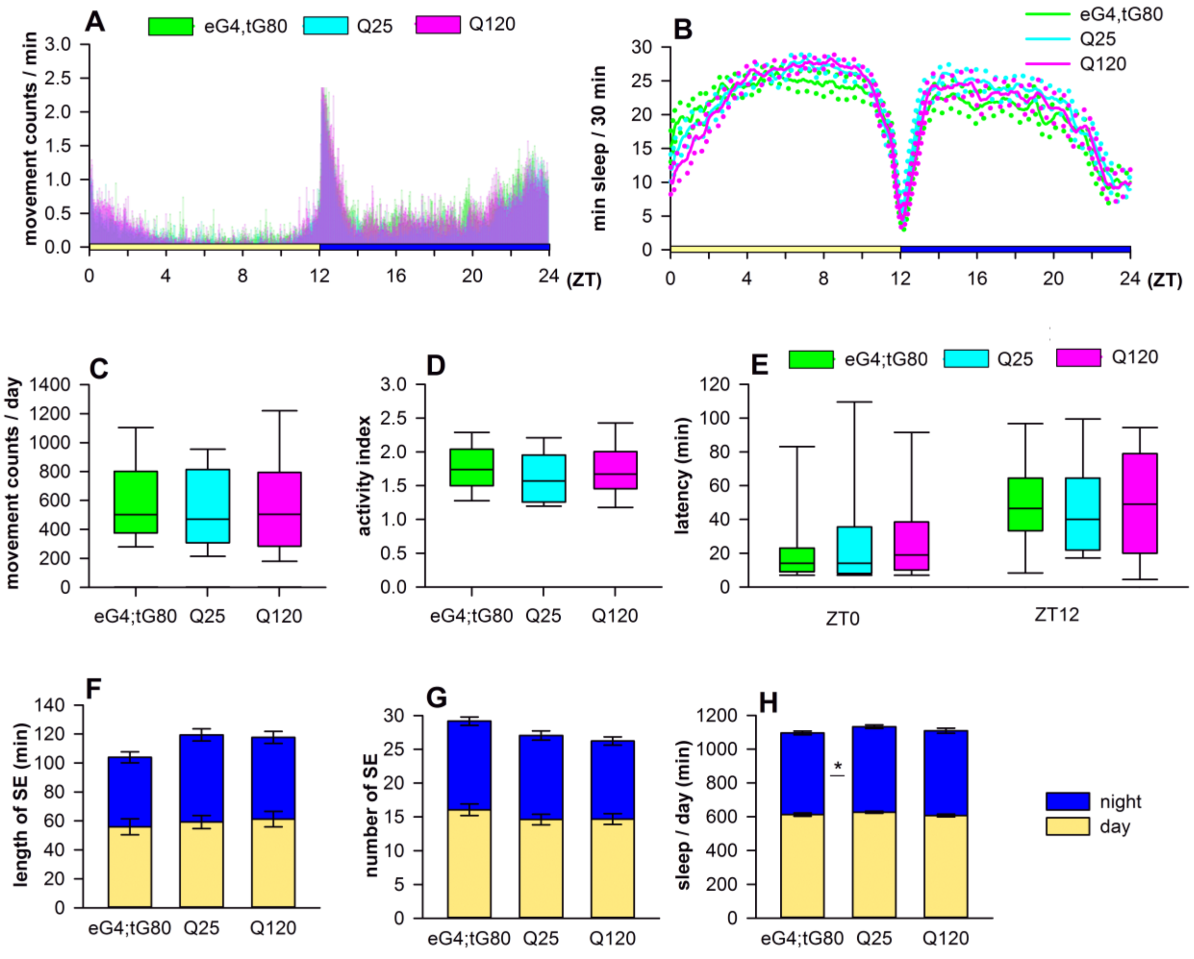

Supplementary Figure S1. Circadian activity and sleep patterns of parental strains.

(A) Daily locomotor activity and (B) sleep patterns (solid lines represent the averages, dotted lines show 95% confidence intervals) of 8 days old flies from the elavGAL4; tubGAL80^{ts} (eG4;tG80), UAS-Httex1Q25 (Q25) and UAS-Httex1Q120 (Q120) parental strains were measured in 12:12 light:dark entrainment. We found no significant differences between these strains in (C) daily movement counts, (D) activity index, (E) sleep latency, (F) length of sleep episodes and (G) number of sleep episodes. (H) The amount of time spent asleep at night was mildly but significantly lower in the eG4,tG80 line than in Q25, however, there was no significant difference between the Q25 and Q120 strains. Data are plotted as mean±SEM (eG4;tG80 n = 87; Q25 n = 91, Q120 n = 98). * $P < 0.05$, one-way ANOVA with Tukey HSD test).
